# Adipose-derived mesenchymal stem cells regenerate radioiodine-induced salivary gland damage in a murine model

**DOI:** 10.1038/s41598-019-51775-9

**Published:** 2019-10-31

**Authors:** Ji Won Kim, Jeong Mi Kim, Mi Eun Choi, Seok-Ki Kim, Young-Mo Kim, Jeong-Seok Choi

**Affiliations:** 1 Department of Otolaryngology, Inha University, College of Medicine, Incheon, Republic of Korea; 2 Department of Nuclear Medicine, National Cancer Center, Goyang, Republic of Korea

**Keywords:** Thyroid cancer, Mesenchymal stem cells

## Abstract

After radioiodine (RI) therapy, patients with thyroid cancer frequently suffer from painful salivary gland (SG) swelling, xerostomia, taste alterations, and oral infections. This study was aimed to determine whether adipose-derived mesenchymal stem cells (AdMSCs) might restore RI-induced SG dysfunction in a murine model. Forty -five mice were divided into three groups; a PBS sham group, a RI+ PBS sham group (0.01 mCi/g mouse, orally), and an RI+AdMSCs (1 × 10^5^ cells/150 uL, intraglandular injection on experimental day 28) treated group. At 16 weeks after RI treatment, body weights, SG weight, salivary flow rates (SFRs), and salivary lag times were measured. Morphologic and histologic examinations and immunohistochemistry (IHC) were performed and the activities of amylase and EGF in saliva were also measured. Changes in salivary ^99m^Tc pertechnetate excretion were followed by SPECT and TUNEL assays were performed. The body and SG weights were similar in the AdMSCs and sham groups. Hematoxylin and eosin staining revealed the AdMSCs group had more mucin-containing acini than the RI group. Furthermore, AdMSCs treatment resulted in tissue remodeling and elevated expressions of epithelial (AQP5) and endothelial (CD31) markers, and increased SFRs. The activities of amylase and EGF were higher in the AdMSCs group than in the RI treated group. ^99m^Tc pertechnetate excretions were similar in the AdMSCs and sham group. Also, TUNEL positive apoptotic cell numbers were less in the AdMSCs group than in the RI group. Local delivery of AdMSCs might regenerate SG damage induced by RI.

## Introduction

Thyroid malignancy is the most common type of endocrine cancer and its incidence has increased constantly over the past years^1^. The treatment of choice for differentiated thyroid carcinoma is surgical resection followed by remnant ablation by radioiodine (RI) therapy. The purposes of RI therapy are; (1) to destroy normal thyroid tissue and increase sensitivity to iodine-131, (2) to remove occult microscopic carcinoma, and (3) to detect persistent carcinoma by iodine-131 total body scanning after RI therapy^2,3^. The mechanism of thyroid tissue destruction is associated with radiation damage through sodium iodide symporter (NIS). However, the expression of NIS gene was observed in the salivary glands (SG), mammary glands, hair, and stomach^4^. During the transport of RI into salivary glands, the mechanism of iodine uptake in the SG is similar to that of the thyroid. Therefore, iodine concentration in SG is 20–100 fold higher than that in serum, and the frequency of RI-induced sialadenitis is cumulative ^131^I dose-dependent^5^. Furthermore, more than 50% of all patients complain of SG dysfunction associated symptoms and have clinically covert xerostomia or post-therapeutic sialadenitis^5^. Actually, SG dysfunction is one of the most frequent chronic complications after RI therapy^6^.

RI-induced SG damage is dose-dependent and irreversible, and high dose RI therapy can induce intermediate or long-term complaints, including sialadenitis, xerostomia, transient taste changes, candida infections, and gingivitis^7,8^. It is difficult to cure RI-induced SG dysfunction due to irreversible damage to acinar, ductal, and endothelial cells^9^. Many researchers have focused on the SG protection and regeneration in the fields of tissue engineering and regenerative medicine, and strategies included autologous SG cells transplantation^10^, the implantation of engineered artificial SG cells^11^, and stem cell therapy^12^.

Mesenchymal stem cells (MSCs) can be isolated from various organ sites including bone marrow, adipose tissue, skin, lung and umbilical cord and differentiate to heterogeneous populations of multipotent stromal cells^13^. Bone marrow-derived MSCs can ameliorate SG damage following irradiation^14^. The densities of MSCs in adipose tissues are higher than those in bone marrow^15^, and adipose-derived mesenchymal stem cells (AdMSCs) maintain the characters of multipotent progenitor cells and capacities for tissue regeneration, trophic effects, and innate immune response^16,17^. For these reasons, AdMSCs have been widely used for tissue restoration and tissue regeneration engineering. Previously, we investigated the effects of the systemic AdMSCs administration on radiation-induced SG damage^18^ and the efficacies of injectable AdMSCs using small intestinal submucosa (SIS) matrices^19^. Several authors have concluded that different types of stem cells possess regenerative activities in the context of external radiation-induced SG damage^13,14,20^. However, no study has yet addressed the regenerative effects or mechanism of AdMSCs on RI-induced SG dysfunction, although the extent of SG damage resulting from RI treatment differs from that caused by external beam radiation^21^.

Here, we examined whether the local injection of AdMSCs into SGs could restore RI-induced salivary dysfunction, and explored the mechanism responsible for the regenerative effects of AdMSCs on RI-induced SG damage.

## Results

### AdMSCs prevented RI induced body weight, SG weight loss, and salivary dysfunction

**Figure 1:**
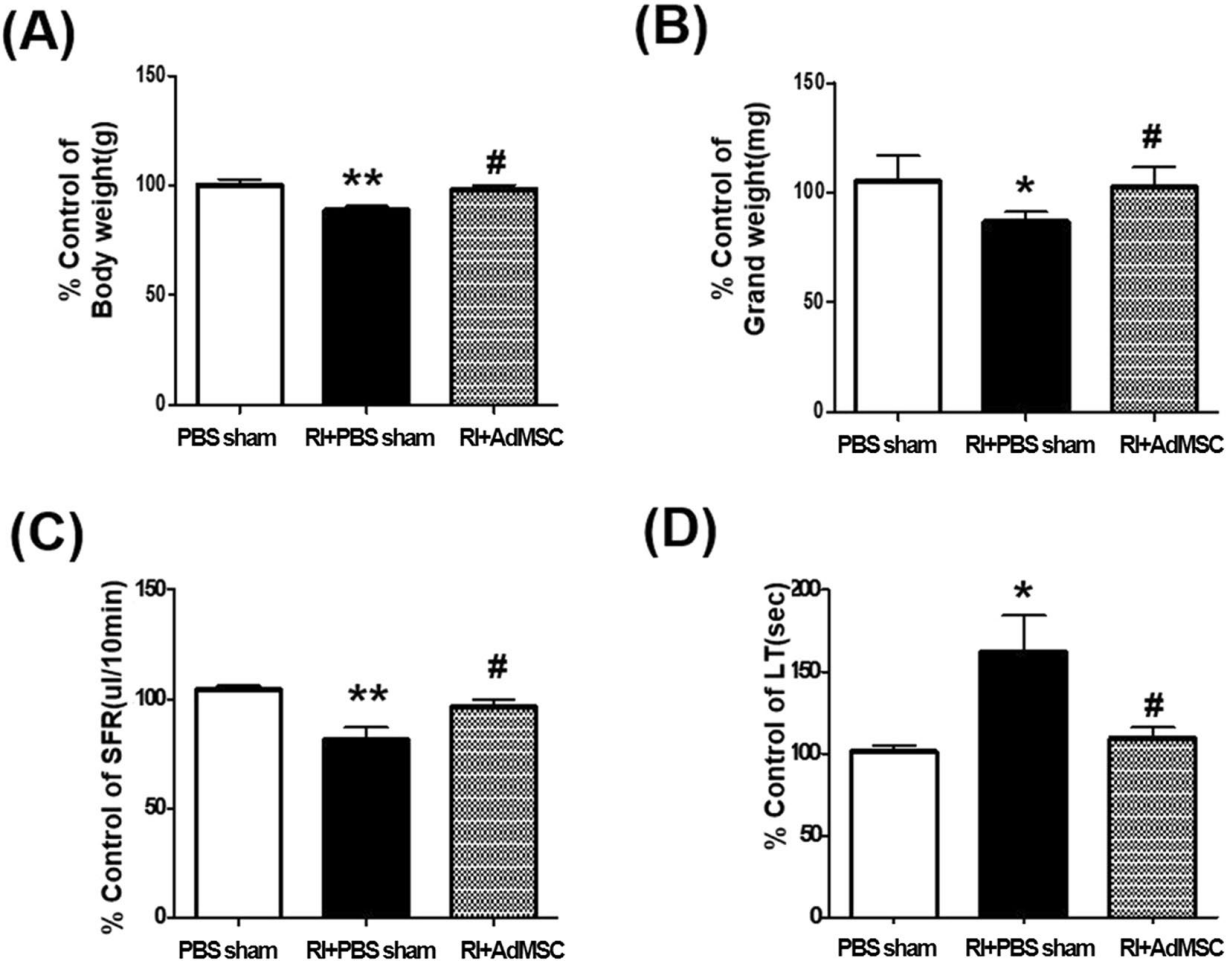
The changes of mice weights (**A**), salivary gland weights (**B**), salivary flow rates (**C**), and salivary lag times (**D**). Results are presented as means ± SEMs. One-way ANOVA, Tukey’s post hoc multiple comparison test. (*compare to PBS sham; ^#^compare to RI + PBS sham, *p < 0.05, **p < 0.01, ^#^p < 0.05). *Abbreviation*: AdMSC, adipose-derived mesenchymal stem cells; PBS, phosphate-buffered saline; RI, radioiodine.

At 16 weeks after RI treatment, mice in the RI + PBS sham group were lighter than the PBS sham group (P < 0.01, Fig. 1A), whereas body weight was preserved in the RI+AdMSCs group. Mean SG weight was also lower in the RI + PBS sham group than in the PBS sham group (p < 0.05, Fig. 1B), but mean SG weights were similar in the RI+AdMSCs and PBS sham groups. To investigate whether AdMSCs injection improved salivary function, SFRs and salivary lag times were measured at 16 weeks after RI treatment. Mean SFR was significantly higher in the RI+AdMSCs group than in the RI + PBS sham group (p < 0.05, Fig. 1C), and mean salivary lag time was significantly lower in the RI+AdMSCs group than in the RI + PBS sham group (p < 0.05, Fig. 1D).

### AdMSCs enhanced SG secretory function

**Figure 2:**
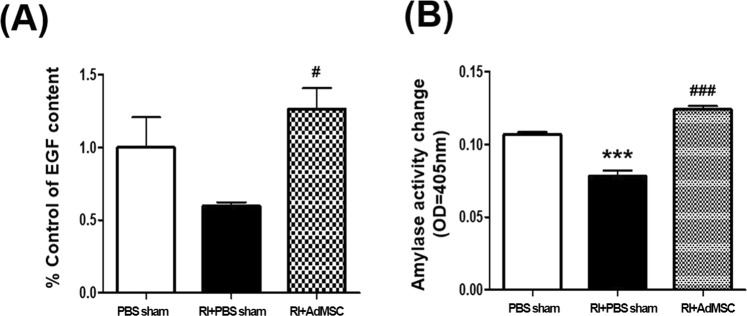
Effect of EGF (**A**) and amylase acivity (**B**). Results are presented as means ± SEMs. One-way ANOVA, Tukey’s post hoc multiple comparison test. (*compare to PBS sham; ^#^compare to RI + PBS sham, ***p < 0.001, ^#^p < 0.05, ^###^p < 0.001). *Abbreviation*: AdMSC, adipose-derived mesenchymal stem cells; PBS, phosphate-buffered saline; RI, radioiodine.

At 16 weeks post-RI, EGF content in saliva was significantly greater in the RI+AdMSCs group than in the RI + PBS sham group (p < 0.05, Fig. 2A), and amylase activity level was also significantly higher in the RI+AdMSCs group than in the RI + PBS sham group (p < 0.05, Fig. 2B).

### AdMSCs ameliorated RI induced histological changes

**Figure 3:**
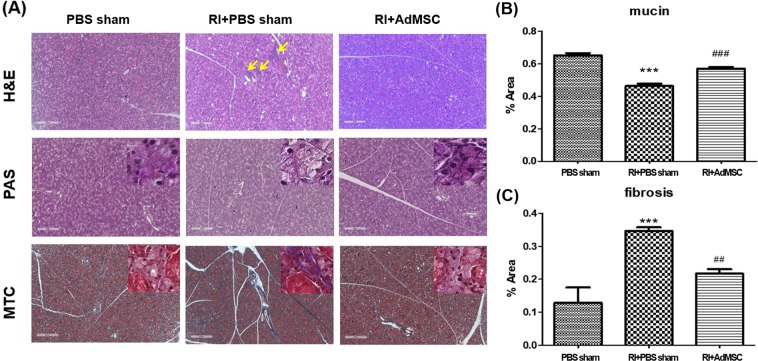
Histological analysis of salivary glands. (**A**) H & E staining revealed few acinar cells and destroyed the ductal structure in the RI + PBS sham group, but these were relatively well maintained in the RI + AdMSCs group (magnification power: x16, Scale bars = 500μm). Arrow represents the areas of inflammatory cell infiltration. (**B**) Mucin-containing acini appeared to be more numerous in the RI + AdMSCs group than in the RI + PBS sham group (**C**) Areas of periductal fibrosis were smaller in the RI+AdMSCs group than in the RI + PBS sham group. Results are presented as means ± SEMs. One-way ANOVA, Tukey’s post hoc multiple comparison test. (*compare to control; ^#^compare to RI + PBS sham, ***p < 0.001, ^##^p < 0.01, ^###^p < 0.001). *Abbreviation*: AdMSC, adipose-derived mesenchymal stem cells; PBS, phosphate-buffered saline; RI, radioiodine; H&E, Hematoxylin and eosin stain; PAS, Periodic Acid Schiff; MTC, Masson’s trichrome stain.

To determine the effects of AdMSCs on RI-induced morphological changes of SGs, we compared microscopic findings of the three. H &E staining revealed the acinar cells with periductal dilatation, fibrosis, and inflammatory cells infiltration in the RI + PBS sham group at 16 weeks post-RI, but the SG parenchyma in the RI+AdMSCs group was well maintained with pure serous acini and ducts. (Fig. 3A) Mucin production was significantly lower in the RI + PBS group than in the PBS sham group and RI + AdMSCs groups as determined by PAS staining (p < 0.05, Fig. 3B). In MTC stained sections, prominent duct dilation and dense fibrous tissue with collagen fibers were observed in the RI + PBS sham group, and these were significantly less dense in the RI+AdMSCs group (p < 0.05, Fig. 3C).

### AdMSCs protected different types of SG cells

**Figure 4:**
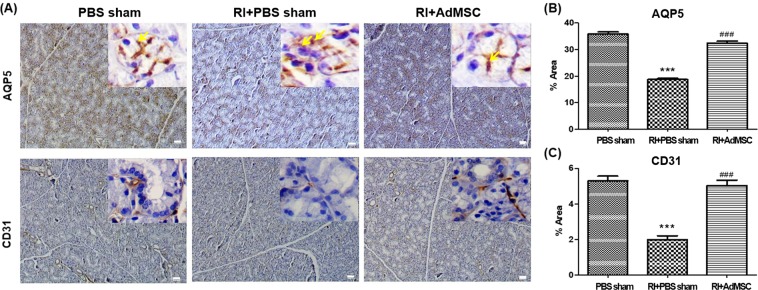
Cytoprotective effects of AdMSCs. (**A**) Immunohistochemistry revealed the expressions of AQP5 (a marker of salivary epithelial cells) and CD31 (an endothelial cell marker) were lower in the RI + PBS sham group than in the RI + AdMSCs and PBS sham groups. (magnification power: x40, Scale bars = 50 μm) Arrow represents the apical localization of AQP5 (magnification power: x200). (**B**,**C**) Quantitative analysis showed the expressions AQP5 and CD31 were greater in the RI + AdMSCs group than in the RI + PBS sham group. Results are presented as means ± SEMs. One-way ANOVA, Tukey’s post hoc multiple comparison test. (*compare to PBS sham; ^#^compare to RI + PBS sham, ***p < 0.001, ^###^p < 0.001). *Abbreviation*: AdMSC, adipose-derived mesenchymal stem cells; PBS, phosphate-buffered saline.

We examined the cellular effects of AdMSCs administration on salivary epithelial, and endothelial cells using phenotypical markers, that is, AQP5 and CD31. Immunohistochemistry revealed that the expressions of AQP5 and CD31 in the RI+AdMSCs and PBS sham group were similar, but only weak positive results were obtained in the RI + PBS sham group (Fig. 4A). In high powered zoom in images represents the apical localization of AQP5. Quantitative analysis showed significantly higher expressions of AQP5 and CD31 (each p < 0.05) in the RI + AdMSCs group than in the RI + PBS sham group (Fig. 4B,C).

### AdMSCs reinstated RI-induced SG dysfunction and apoptosis

**Figure 5:**
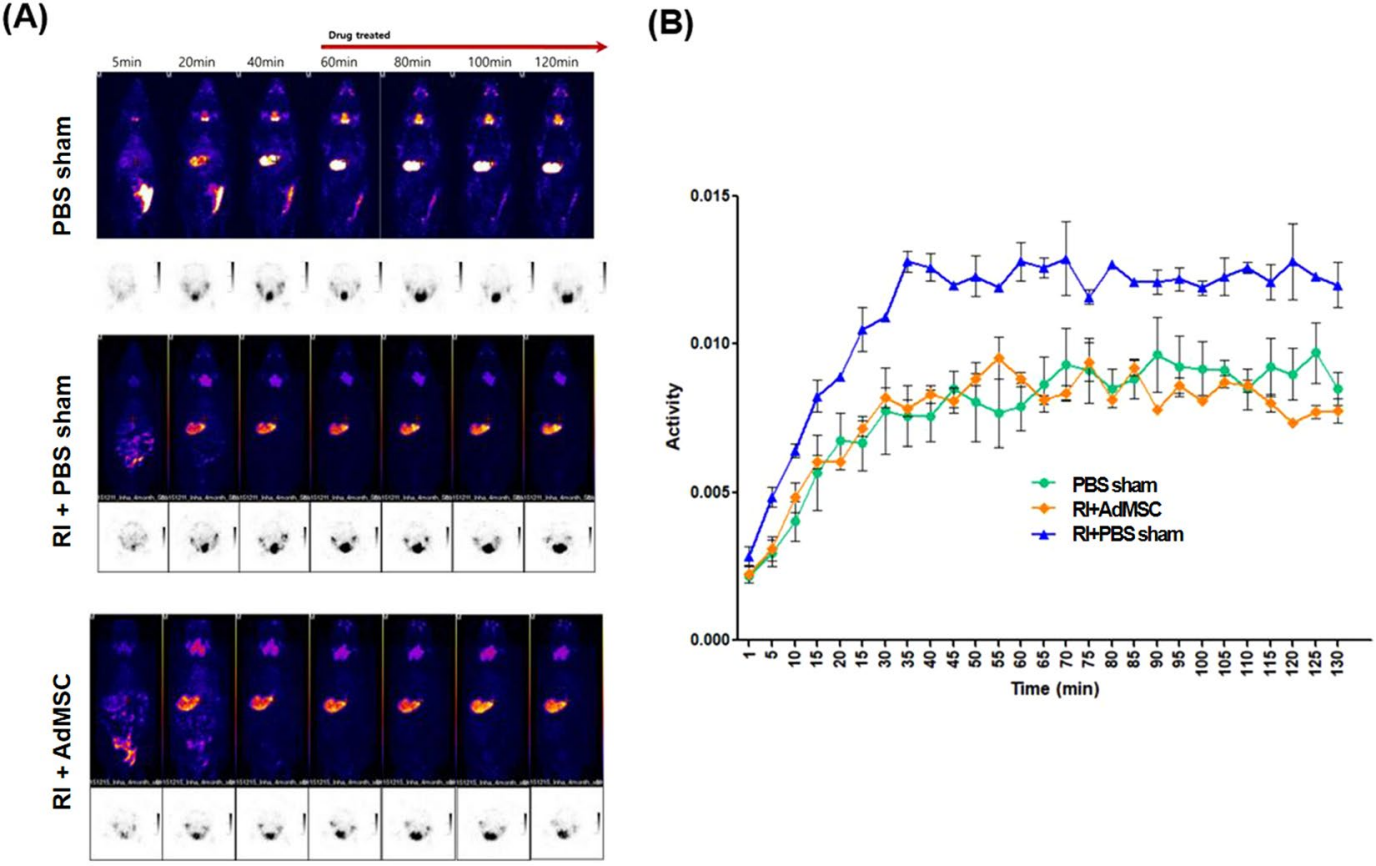
(**A**) Single photon emission computed tomography (SPECT) image (**B**) Dynamics of ^99m^Tc pertechnetate after RI. ^99m^Tc pertechnetate excretion was markedly lower in the RI + PBS sham group than in the RI + AdMSCs and PBS sham group, in which ^99m^Tc pertechnetate excretions were similar. *Abbreviation*: AdMSC, adipose-derived mesenchymal stem cells; PBS, phosphate-buffered saline; RI, radioiodine.

**Figure 6:**
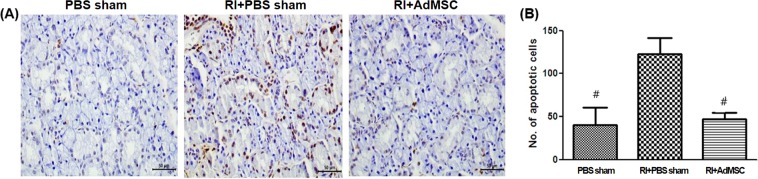
Anti-apoptotic effects of AdMSCs on salivary glands. (**A**) TUNEL analysis image at 16 weeks after AdMSC transplantation. (**B**) Quantified TUNEL positive apoptotic cells count. The number of apoptotic cells in RI + PBS sham group is higher than that of RI+AdMSCs and PBS sham group. Results are presented as means ± SEMs. One-way ANOVA, Tukey’s post hoc multiple comparison test. (*Compare to PBS sham ^#^compare to RI + PBS treated, *p < 0.05). *Abbreviation*: AdMSC, adipose-derived mesenchymal stem cells; PBS, phosphate-buffered saline; RI, radioiodine; TUNEL,Terminal deoxynucleotidyl transferase dUTP nick end labeling.

In order to evaluate the excretory function of submandibular glands, pilocarpine was administered at 60 minutes after ^99m^Tc pertechnetate injection to induce the excretion of ^99m^Tc pertechnetate through the saliva from the submandibular glands. ^99m^Tc pertechnetate excretion was markedly lower in the RI + PBS sham group than in the other two groups and was similar in the RI + AdMSCs and PBS sham groups (Fig. 5A,B). In TUNEL assay, TUNEL-positive apoptotic cell numbers in the RI + AdMSCs group were significantly lower than in the RI + PBS sham group (Fig. 6A,B).

### Stem Cell Engraftment into the salivary gland after transplantation

PCR analysis indicated the presence of hALU mRNA in the AdMSC group at 16 weeks. It suggests that AdMSCs in salivary gland were fostered to differentiate into progenitor cells (Supplementary Fig. 1). Fluorescent *in situ* hybridization analysis revealed FITC–labeled AdMSCs in the RI + AdMSC group at 16 weeks after transplantation (Supplementary Fig. 2).

## Discussion

This study confirms the reparative and regenerative effects of injectable AdMSCs in a preclinical setting. These effects were first described by Saylam *et al*., who showed systemic AdMSCs administration ameliorated RI-induced histologic changes and salivary dysfunction in a rat model^17^. Here, we showed locally injected AdMSCs enhance SG secretory function after RI treatment by attenuating or preventing RI-induced damage. A dose of 0.01 mCi/g RI was administered to animals and they received an AdMSCs injection into the submandibular gland 4 weeks after RI treatment. RI dose was determined based on a review of related animal studies. We verified the thyroid tissue was completely ablated 14 days after 0.01 mCi/g RI treatment on SPECT, which suggests that the dose and time of RI for ablation were appropriate showing a similar effect on surgical thyroidectomy. In particular, this study demonstrates the regenerative effects of local AdMSCs administration on SG dysfunction after RI treatment in a preclinical context.

In the present study, significant intergroup differences were observed for body and SG weights. In a previous study, RI exposed SGs showed atrophy of parenchyma, tissue necrosis, and fibrosis resulting from inflammation^17^, and in another, it was suggested simultaneous AdMSCs transplantation might protect SG cells from atrophied and act to regenerate acinar cells and restore saliva volume^22^. In the present study, salivary lag times were shorter and salivary flow rates were significantly higher in the RI + AdMSCs group than in the RI + PBS sham group, which also suggests AdMSCs protect SG cells against RI.

Although the paracrine actions of the stem and progenitor cells are now known to play roles in wound healing, the entirety of biomolecule production by various progenitor cell populations and their roles in wound healing have yet to be characterized^23^. Our results suggest that AdMSCs might secrete paracrine factors, such as EGF, and maintain amylase secretory function. Ductal cells contain more NIS protein in the membranes than those of acinar cells^24^, but acinar cells are destroyed by RI by an unknown mechanism^21^. Prendes *et al*. hypothesized that RI injury to ductal and acinar cells occurs secondary to damage to endothelial cells in SGs^25^. In the present study, we observed diffuse endothelial cell destruction and fibrosis at 16 weeks post-RI, which was supported by a reduction in the expression of CD31 (an endothelial marker), and is in-line with previous reports of saliva reductions and reduced SPECT excretion^26^. In the present study, the acinar cells with periductal dilatation, fibrosis, and inflammatory cells infiltration in the RI + PBS sham group at 16 weeks post-RI. This is consistent with previous study^17^ using RI to induce salivary dysfunction. They showed the histological finding including the epithelial edema, vacuolization, periacinar inflammation, ductal necrosis, ectasia, periductal fibrosis and vascular sclerosis after 1 and 6 months after RI treatment. We could identify the lymphocytic infiltration in RI + PBS sham group. However, decreased inflammatory cell infiltration were shown in AdMSCs administered salivary gland. RI induced sialadenitis could decrease salivary functions. In our study, the activity of ^99m^Tc pertechnetate remained high after pilocarpine administration in the submandibular gland of the RI + PBS sham group in compared with the other two groups. This implies that ^99m^Tc pertechnetate is constantly concentrated in the submandibular gland due to a functional secretory defect in the RI + PBS sham group. Moreover, the activity of ^99m^Tc pertechnetate in the RI+AdMSCs was similar to the PBS sham group.

Redman RS showed that the radiation-induced stressful events on SG cells could replace and regenerate themselves the differentiated SG cells of the acini and large ducts of mature, but after heavy doses (more than 6000 rads) of radiation, the serous acini of SG nearly disappear and the regeneration decrease^27^. ^131^I causes acute cell death by emitting short path-length (1 to 2 mm) beta particles^28^, and RI induced hypoxic damage or reactive oxygen species (ROS) production modulates the release of soluble paracrine factors by stem cells and progenitor cells. Furthermore, paracrine mediators like growth factors, have been reported to provide acute radioprotection, to have proliferative, cytoprotective and angiogenic effects, to promote cell migration and extracellular matrix homeostasis, and to decrease fibrosis and apoptosis. The reduced apoptosis observed in the AdMSCs group in the present study, was thus, probably related to the paracrine actions of AdMSCs. These findings are in accordance with those of earlier studies^14,19,26^.

Konings *et al*.^29^. suggested unique early and late phases of salivary dysfunction after exposure to radiation. During the early or acute phase, cellular functions are disrupted by membrane damage, especially endothelial damage, whereas late or delayed phase effects are caused by radiation killing of progenitor cells and damage to the cellular environment. Bioactive factors like EGF are released from acinar cells stimulated by AdMSCs and help maintain SG functions^23^. Also, progenitor and stem cells increase host cell survival in ischemic environments and enhance the paracrine effects of stem cell, and thus provide functional benefits during the delayed and late phases^30^.

The mechanism and treatment of radiation-induced damage are reasonably well understood, whereas those of RI-induced sialadenitis are not. Maier *et al*. concluded that the inhomogeneous distribution of ^131^I activity in SGs probably causes isolated regions of greater damage than that caused by external beam radiation^28^. In SGs, ^131^I has a greater effect on parotid serous cells than to submandibular gland mucus-secreting acinar cells. Nagler *et al*.^31^ theorized that delayed serous cell death leading to specific parotid radiosensitivity might be due to the redox effects of metal ions, such as, iron and copper, in secretion granules. Jeong *et al*.^6^ reported that patients who underwent thyroidectomy for thyroid cancer had parotid glands susceptible to RI-induced sialadenitis, and that xerostomia symptoms were associated with submandibular gland dysfunction and the prevalence of dysfunctional salivary glands. In this study, we identified similar uptakes in parotid and submandibular glands after RI therapy, though for convenience, we harvested only submandibular glands.

This study has some limitations that warrant mention. We did not observe significant transdifferentiation from AdMSCs to SG cells, and did not attempt to measure the extent of SG cell replacement by transdifferentiation. However, it was recently suggested that the beneficial effects of stem cells might be due to their paracrine activities as wells as their abilities to differentiate^23^. In the present study, we found the intraglandular delivery of AdMSCs in mice effectively prevented RI-induced salivary cell death and structural remodeling and induced to the release of bioactive factors like EGF by acinar cells. However, further investigations are necessary to identify the specific factors secreted by AdMSCs that contribute to remodeling and cell survival after RI damage. Furthermore, total saliva volume is made from all salivary glands including parotid, submandibular, and sublingual glands. However we did AdMSCs submandibular gland intraglandular injection. Therefore, total saliva production was underestimated even though the saliva volume from the parotid gland is small. Saylam, G. *et al*. demonstrated that systemic administration of AdMSCs could play a promising role as a protective/regenerative agent against RAI-induced salivary gland dysfunction. In the future, the studies about the effects comparison between systemic administration vs. local injection of AdMSCs is needed. Lastly, there were not the FISH data at 2 and 4 week time point. However, we can identify that mesenchymal stem cells are present at 16 weeks, that suggests that the survival of AdMSC in salivary gland tissue during the experimental time.

In conclusion, this study indicates the intraglandular delivery of AdMSCs in mice protects against RI-induced SG damage and has paracrine effects that promote tissue restoration. We believe that AdMSCs should be considered candidates for cell-based therapies targeting RI-induced SG hypofunction in thyroid cancer patients.

## Materials and Methods

### Preparation of AdMSCs

We utilized surplus frozen AdMSCs from a cell bank (K-Stem Cell, Seoul, Korea). Patients had agreed the use of these cells with the written informed consent for the purpose of research. The characteristics of AdMSCs were consistent with usual mesenchymal stem cells and compatible with published data using the same protocols^19,30^. Shortly, subcutaneous fat tissues were dissolved with 4 ml of RTase cell isolation enzyme (K-Stem Cell, Seoul, Korea) per one gram of adipose tissue at 37 °C for 60 minutes. The dissolved materials were refined through a 100-μm filter to discard the cellular detritus and undergone with the centrifugation for 5 minutes at 1,500 rpm. The upper layer was resuspended in K-Stem Cell media containing 10% FBS for the MSC attachment. After secondary centrifugation at same speed and duration, the pellet were cultured at 37 °C overnight. Cell adhesion was examined for 24 hours. Adherent cells were washed with PBS and the medium changed to 5% FBS containing RKCM (RNL Bio media for AdMSC culture). The cell culture were continued for 4–5 days until passage 3 at 90% confluence^30^. The immunophenotypes of the AdMSCs were examined using a FACS (fluorescence-activated cell sorting) and CellQuest software (Becton Dickinson, San Jose, CA). Harvested AdMSCs showed a homogeneous cell population containing the characteristics of MSCs^30^.

### RI induced salivary damage in the thyroid-ablated animal model

Four-week-old female C57BL/6 mice were purchased from Research Model Producing Center Co. Ltd. (Orient Bio, Seongnam, Korea). All animal experimental protocols were approved by the Institutional Animal Care and Use Committee (IACUC) of the National Cancer Center and Inha University (INHA 180402-540) and performed after obtaining approval (NCC-13-186D). All methods were performed in accordance with relevant guidelines, regulations through the international standards of animal protection and the ethical principles of the National Council of Animal Experimentation. Animals were randomly allocated to three groups; a PBS sham group (n = 15), a RI + PBS sham group (n = 15), and a RI + AdMSCs group (n = 15). RI was administered orally at a dose of 0.01 mCi/g to animals in the RI + PBS sham and RI + AdMSCs groups. Mice in the RI + AdMSCs group received an AdMSCs injection into the submandibular gland 4 weeks after RI treatment (experimental day 28; ED28). Briefly, a neck incision was performed to expose submandibular glands, and then either 1 × 10^5^ AdMSCs in 150 µl of PBS or PBS alone (RI + PBS sham group) were injected directly to both glands using a syringe equipped with a 25-gauge needle. On single photon emission computed tomography (SPECT) planar whole-body and neck images obtained for identifying the immediate secretion function after ^99m^Tc pertechnetate injection and repeated every 5 minutes about 1.5 hour at ED112. RI treated mice showed RI accumulation around parotid and submandibular glands. The thyroid was not imaged by neck transaxial planar imaging in RI-exposed mice due to the ablative effects of treatment at ED7. Animals were administered 1.5 μg/100 g of thyroxine in drinking water to maintain an euthyroid state^32^.

### Polymerase chain reaction (PCR) for the human ALU (hALU) gene

DNA was extracted from the tissues using the DNeasy Blood & Tissue kit (QIAGEN, Valencia, CA, USA). PCR amplification of hALU was undergone using AccuPower PCR PreMix (Bioneer, Korea) under the conditions: 94 °C for 5 minutes, 40 amplification cycles (94 °C, 55 °C, and 72 °C for each 1 minute), followed by expansion for 10 minutes at 72 °C. The primers CAGGACCTGAGAAAGGACACTATCC (forward) and CAAACAAGAGGCA CACTTT CAACCA (reverse) were used^19^. The intensity of PCR product were measured using an image analysis program (Image J, National Institute of Health, Bethesda, MD, USA).

### Fluorescent *In Situ* Hybridization (FISH) analysis

We performed the FISH analysis using a Biotin (FITC) detection kit (1089-KB-0, Cambio, UK). Sectioned tissue slides were deparaffinized, hydrated again and incubated with sodium thiocyanate solution. Then, they were incubated in pepsin solution for 20 minutes at 37 °C followed by PBS washing. Tissues were fixed in paraformaldehyde for 2 minutes, followed by rinsing with PBS, and dehydration. Cellular DNA was denatured by immersing slide in 70% formamide at 70 °C for 2 minutes. The probe was denatured 80 °C for 10 min and added to denatured tissue slides and hybridized for overnight at 37 °C humidified chamber. After washing for 5 minutes in wash solution, the slides were incubated in detergent wash solution for 15 minutes at 37 °C, washed again with PBS. Nuclei were DAPI-counterstained and we acquired a confocal laser-scanning microscope imagings (Olympus FV 1000, Japan).

### Assessment of body weights, SG weights, salivary lag times, and flow rates

Body weights were measured at 16 weeks after RI administration and saliva was collected from mouth floors using a micropipette 5 minutes after muscarinic cholinergic agonist pilocarpine (2 mg/kg i.p.). Acetylcholine is responsible for causing the salivary glands to make saliva stimulation. Salivary flow rates (SFRs) and lag times were measured at 16 weeks after RI treatment. Salivary lag times were calculated from SG stimulus to the beginning of saliva excretion. Mice were euthanized 16 weeks after RI treatment. Submandibular glands were harvested and surrounding fat and connective tissues were removed. The weights of both submandibular glands in each animal were measured before formalin buffer fixation and paraffin embedding.

### Measurement of epidermal growth factor (EGF) and amylase levels in saliva

Saliva samples was collected at 16 weeks post-RI treatment. We measured the saliva EGF using an enzyme-linked immunosorbent assay (ELISA) kit product (Quantikine; R&D Systems, Minneapolis, MN, USA). We examined activity of amylase in the saliva using α-amylase assay kit (Salimetrics LLC, State College, PA, USA). The amount of α -amylase activity is directly comparable to absorption raise at 405 nm.

### Morphology analysis and immunohistochemistry study

Submandibular gland tissue sections were stained with Hematoxylin & Eosin (H&E), periodic acid-Schiff (PAS; Sigma- Aldrich), and Masson’s trichrome (MTC). The ratios of surface areas occupied by PAS-positive cells to total measured areas were calculated for five random fields at X400 under a light microscope. Assessments were performed in 3 randomly selected sections by two independent observers using ImageJ software (National Institutes of Health, Bethesda, MD). The immunohistochemical study was performed using the following antibodies: anti-aquaporin 5 (anti-AQP5; 1:200; Alomone Labs, Jerusalem, Israel); anti-cluster of differentiation 31 (anti-CD31; 1:50; Abcam, Cambridge, UK)^19^. Examiner evaluated three fields per section randomly, and selected areas were anaylyzed using the Metamorph software (Molecular Devices Corporation, Sunnyvale, CA, USA).

### Terminal deoxynucleotidyl transferase biotin-dUTP nick end labeling (TUNEL) assay

Apoptotic cells in SGs were identified using a TUNEL assay (ApopTag Plus *in situ* Apoptosis Kit; Chemicon Int., Temecula, CA, USA) at 16 weeks post-RI exposure. TUNEL-positive apoptotic cells at x400 and the numbers of cell in apoptosis were calculated in ten random sites^32^.

### SPECT image analysis

SPECT images were processed using Osirix imaging software (The Osirix Foundation, Geneva, Switzerland) and InVivoScope (Bioscan Inc). We measured the regions of interest (ROIs) around the thyroid manually and obtained the SGs images 60 minutes after ^99m^Tc pertechnetate injection. ROIs were compose of a volume of interest (VOI), and VOIs were pasted into SPECT images. All VOIs need the correction the noise counts from adjacent tissues like bone^32^. The radioactivities of all voxels in VOIs were calculated for activity degradation posttreatment. Maximal normalized radioactivities in VOIs were adopted as representative values to lessen partial volume effects^32^.

### Statistical analysis

The statistical analysis was performed using the GraphPad Prism 5 package (GraphPad Software Inc, La Jolla, CA). Comparisons among three groups were performed using one way ANOVA followed by Tukey’s post hoc test, and paired group comparisons were performed using two-way ANOVA followed by Bonferroni’s post hoc test. Statistical significance was accepted for p values < 0.05.

## Supplementary information

human(h)ALU mRNA PCR and FISH data

## Data Availability

All data generated or analyzed during this study are included in this published article.
