# Supplementary material for: Adipose-derived mesenchymal stem cells regenerate radioiodine-induced salivary gland damage in a murine model
Source: Sci Rep. 2019 Oct 31;9:15752. doi: 10.1038/s41598-019-51775-9 (PMC6823479; doi:10.1038/s41598-019-51775-9)
Supplement: Supplementary file 1 — human(h)ALU mRNA PCR and FISH data [file 41598_2019_51775_MOESM1_ESM.docx]

**SUPPLEMENTARY INFORMATION**

***Adipose-derived mesenchymal stem cells regenerate radioiodine-induced salivary gland damage in a murine model***

*Ji Won Kim^a^, Jeong Mi Kim^a^, Mi Eun Choi^a^, Seok-Ki Kim^b^, Young-Mo Kim^a^ and Jeong-Seok Choi^a,*^*

*^a^Department of Otolaryngology, Inha University, College of Medicine, Incheon, Republic of Korea*

*^b^Department of Nuclear Medicine, National Cancer Center, Goyang, Republic of Korea*

****Correspondence to*** *Jeong-Seok Choi, MD, Ph D*

*^a^Department of Otorhinolaryngology, Inha University College of Medicine, 27 Inhang-ro, Jung-gu, Incheon, Republic of Korea, 22332*

*Phone: +82-32-890-3570; Fax: +82-32-890-3580; E-mail: jschoi@inha.ac.kr*


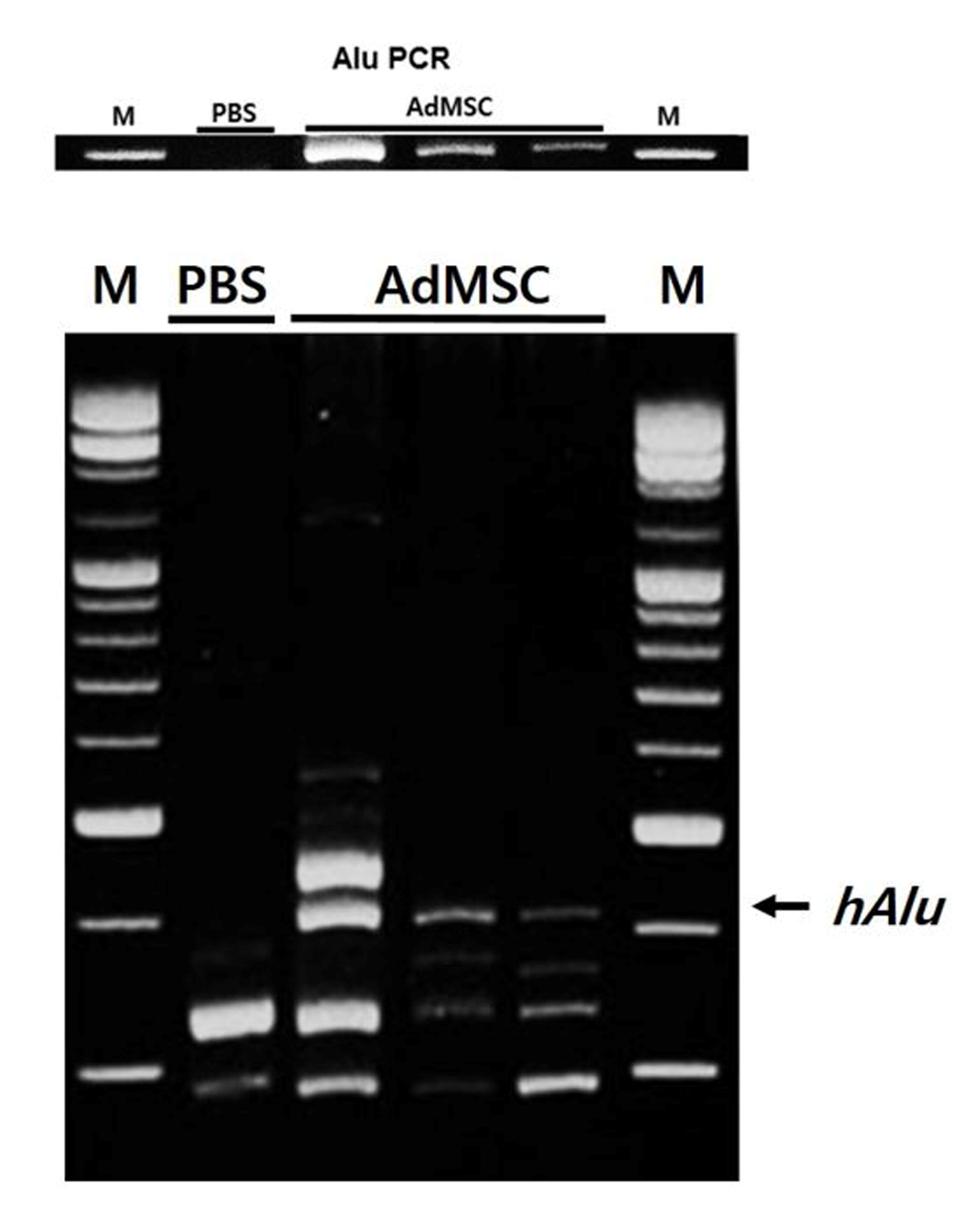
**Supplementary figure 1.** Cropped images of polymerase chain reaction (PCR) analysis of human(h)ALU mRNA (M, marker; PBS, RI + PBS sham group; AdMSC, RI + AdMSC group)


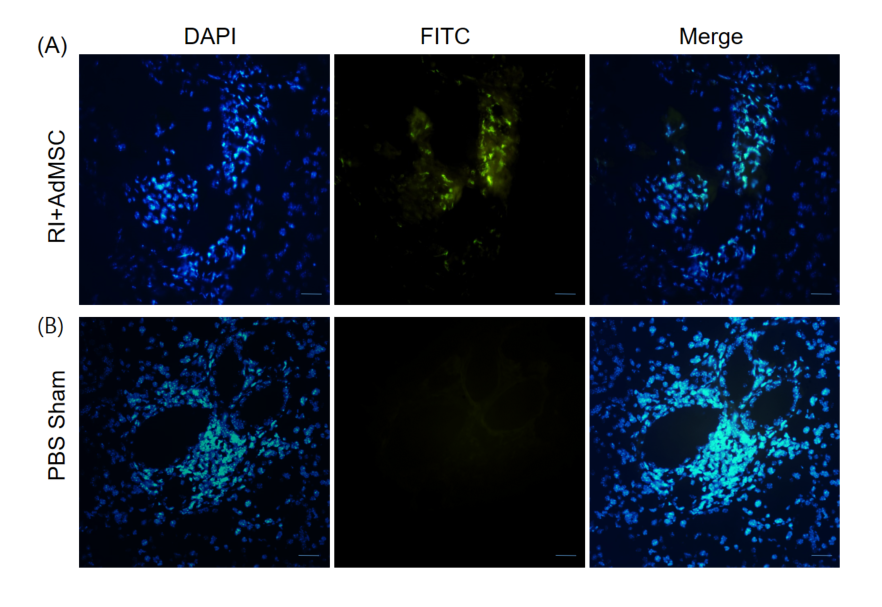


**Supplementary figure 2.** At 16 weeks after transplantation, (A) RI + AdMSCs groups showed FISH positive cells in salivary gland unlike (B) PBS sham group. Bar = 50 μm.

*Abbreviation*: DAPI (4′,6-diamidino-2-phenylindole); FITC, Fluorescein isothiocyanate; RI, radioiodine; AdMSCs, adipose‐derived mesenchymal stem cells; FISH, Fluorescent *In Situ* hybridization
